# Intrathoracic Humeral Head Fracture: A Case Report

**DOI:** 10.7759/cureus.86554

**Published:** 2025-06-22

**Authors:** Kalvin Zee, Shiv Patel, Joseph Losh

**Affiliations:** 1 General Surgery, MercyOne Medical Center, Des Moines, USA; 2 Acute Care Surgery, MercyOne Medical Center, Des Moines, USA

**Keywords:** hemopneumothorax, intrathoracic humeral head fracture, thoracotomy, trauma, traumatic aortic injury

## Abstract

An intrathoracic humeral head fracture is a rare injury usually caused by a high-energy force to the shoulder, resulting in medial displacement of the humeral head. This injury can be severe and life-threatening due to the proximity of the dislocated and fractured bone to the critical structures within the intrathoracic cavity. There are no current official guidelines for the treatment of such injuries. We report the successful treatment of a patient with an intrathoracic humeral head fracture that lacerated the thoracic aorta.

## Introduction

An intrathoracic humeral head fracture is an extremely rare injury usually caused by a high-energy force to the shoulder, resulting in medial displacement of the humeral head, first described in 1949 [1]. This injury can be severe and life-threatening due to the proximity of dislocated and fractured bone to the critical structures within the intrathoracic cavity [2]. There are no current official guidelines for the management of such injuries. We report the successful treatment of a patient with an intrathoracic humeral head fracture that lacerated the thoracic aorta.

## Case presentation

**Figure 1:**
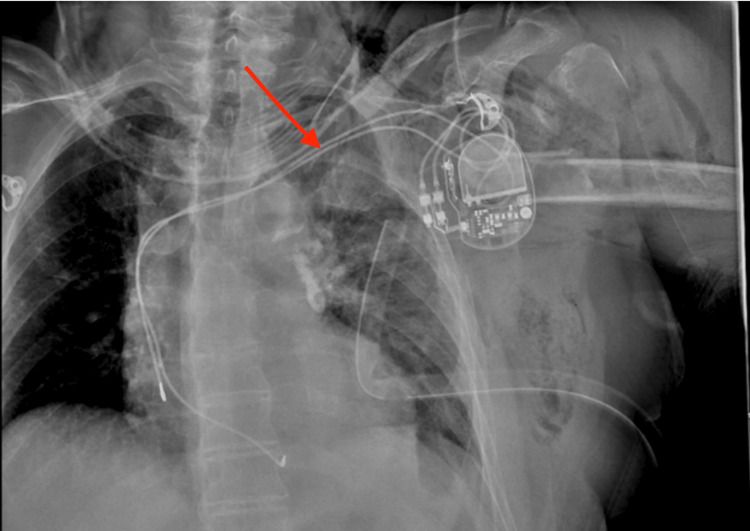
X-ray showing the intrathoracic humeral head fracture (red arrow)

**Figure 2:**
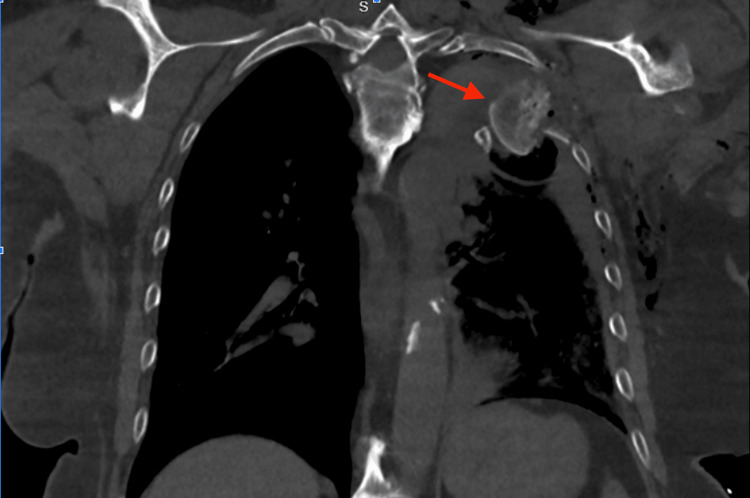
CT in coronal view showing the comminuted left humeral fracture with fragments of the humeral head penetrating through the left chest wall and pleura (red arrow)

An 81-year-old female presented as a trauma alert after suffering a fall off her lawn mower. The patient’s primary survey was significant for left-sided diminished breath sounds with normal blood pressure of 111/59 mmHg. A chest tube was placed in the trauma bay with the return of 600 mL of blood and air. On further examination, pain was noted in the upper left chest with associated deformity of the left arm. The chest X-ray showed a left humeral metaphyseal fracture with a portion of the humeral head penetrating the left chest wall, fractures of ribs two through four, and left-sided hemopneumothorax (Figure 1). The patient remained hemodynamically stable throughout this assessment, so a computed tomography (CT) scan was performed, showing a comminuted left humeral fracture with fragments of the humeral head penetrating through the left chest wall and pleura (Figure 2). Given the concern for major vessel injury due to the proximity of bone fragments, the patient was transported to the intensive care unit (ICU) for close monitoring. There were no immediate plans for operative intervention given her stable vital signs.

The patient was recovering as expected until hospital day three, when she became acutely hypotensive with tachycardia and dyspnea. There was also a significant increase in chest tube output at this time from 35 mL/hr to 200 mL/hr in the last hour. Shortly after, the patient lost pulses and required three rounds of cardiopulmonary resuscitation (CPR) before return of spontaneous circulation (ROSC). During CPR, her chest tube output increased to 1500 mL. A massive transfusion protocol was initiated, cardiothoracic surgery was contacted, and the patient was taken emergently to the operating room.

A standard left-sided posterolateral thoracotomy was performed. The left fourth intercostal space was opened, and significant hemothorax was encountered. The humeral head and other bone shards were removed from the thoracic cavity. A 5 mm laceration of the anterolateral descending thoracic aorta was found with active bleeding. This was repaired with a 4-0 polypropylene pledgeted suture. The patient’s hemodynamics subsequently improved with ongoing transfusion. A significant lung contusion with associated laceration was also encountered. This was also repaired with a 4-0 polypropylene with subsequent hemostasis. The patient remained stable on low-dose vasopressor support, and the left chest cavity was washed out and closed, and the patient was transferred back to the ICU postoperatively. 

The patient was extubated on postoperative day (POD) 3 and transferred out of the ICU on POD 5. The chest tube was placed to water seal on POD 5 and removed on POD 6. Postoperative care was complicated by a superficial wound infection requiring debridement and reaccumulating fluid in the chest requiring repeat drainage. Overall, she fared well and was ultimately discharged to a rehabilitation facility. An orthopedic intervention was deferred during this hospital admission due to the infection, with future follow-up for reverse shoulder arthroplasty.

## Discussion

An intrathoracic fracture of the humeral head is a very rare occurrence that is usually the result of high-energy traumas to the shoulder, resulting in medial displacement of the humeral head. It is generally associated with high-energy traumas involving the shoulder due to falls, bike accidents, or motor vehicle collisions [1]. The mechanism of injury was first proposed by Hardcastle et. al in 1981, which involves two phases. First, a fall in abduction and external rotation of the arm causes dislocation and medial displacement of the humeral head towards the chest. Then, a sharp adduction results in a humeral head fracture [2]. A sudden high-energy force is usually needed to fracture both the humerus and drive the dislocated humeral head into the chest wall and mediastinum [3].

Only 28 cases have been reported since West first described this injury in 1949 [4,5]. Due to the rarity of this event and the low number of cases reported, there is no current guideline or standard for treatment [3]. However, this injury can cause severe, life-threatening conditions due to the proximity of the humeral head to the thorax and surrounding structures. 

Intrathoracic displacement of the humeral head results in a myriad of injuries. Virtually every injury has been accompanied by a pneumothorax, hemothorax, or both with associated pulmonary contusions [1,6]. Additionally, aortic compression, aortic injury, subclavian artery injury, and costocervical trunk injuries have been described with intrathoracic displacements of the humeral head [1]. Finally, neurologic injuries can occur, including brachial plexus lesions [1]. In the case of our patient, no neurologic injuries were sustained with the injury.

Due to the rare nature of this injury, there is no current consensus on the treatment of patients with intrathoracic fractures of the humeral head. Treatment has ranged from conservative measures with volume resuscitation and chest tube placement to thoracotomy, depending on the severity of the injury. We recommend that hemodynamically stable patients undergo a CT angiography to further delineate major vessel involvement, with prompt vascular assessment should intervention be warranted. Additionally, early operative intervention with cardiothoracic surgery should be pursued to remove intrathoracic bone shards, thereby preventing the laceration of major vessels. 

Reconstruction of the humerus has been done by the implantation of anatomical or reverse prostheses [7], with the goal of anatomical reconstruction for younger patients. Hemiarthroplasty has also been an appropriate alternative [4]. In elderly patients, open reduction internal fixation (ORIF) has been recommended when vascular integrity is in doubt, preventing avascular necrosis of the humeral head [8].

One-year postoperative range of motion outcomes have been varied [4]. Full range of motion return has been documented [9], as has decreased range of motion outcomes [3,10]. In rare instances, avascular necrosis of the humeral head has occurred, leading to a reoperation [2].

## Conclusions

Intrathoracic humeral head fractures can cause devastating cardiovascular injuries, and identification of injuries must be undertaken immediately. While there are no current guidelines to treat intrathoracic humeral head fractures, in this case, surgery was necessitated due to the thoracic aortic injury.

CT angiography should be performed for hemodynamically stable patients with intrathoracic humeral head fractures to identify any possible aortic injury due to bone fragments and assist with operative planning for reconstruction. Earlier identification of this injury can help prevent the need for emergent thoracotomy.
